# A new species of *Ovabunda* (Octocorallia, Xeniidae) from the Andaman Sea, Thailand with notes on the biogeography of this genus

**DOI:** 10.3897/zookeys.431.7751

**Published:** 2014-08-05

**Authors:** Michael P. Janes, Catherine S. McFadden, Thanongsak Chanmethakul

**Affiliations:** 1 AquaTouch, 12040 North 32nd Street, Phoenix, Arizona 85028, USA; 2 Harvey Mudd College, Department of Biology, 1250 North Dartmouth Avenue, Claremont, California 91711, USA; 3 Phuket Rajabhat University, Department of Applied Biology, Faculty of Science and Technology, Phuket, Thailand 83000

**Keywords:** Cnidaria, Coelenterata, phylogeny, sclerites, SEM, soft coral, taxonomy

## Abstract

A survey of xeniid octocorals was carried out in the waters off Southwestern Thailand in September, 2007. Microscopic investigation of the colonies revealed that three specimens belonged to the genus *Ovabunda*. Gross morphological examination is presented here accompanied by scanning electron micrographs of the sclerites. Molecular phylogenetic analysis showed identical genotypes at *mtMutS*, *COI*, and 28S rDNA for all three specimens and supports their generic assignment. Colony size and shape, sclerite size, and pinnule arrangement differ from nominal species of *Ovabunda* and thus a new species, *O. andamanensis* is introduced here. This work also presents a new eastern geographical record for the genus *Ovabunda*.

## Introduction

**Figure 1.**
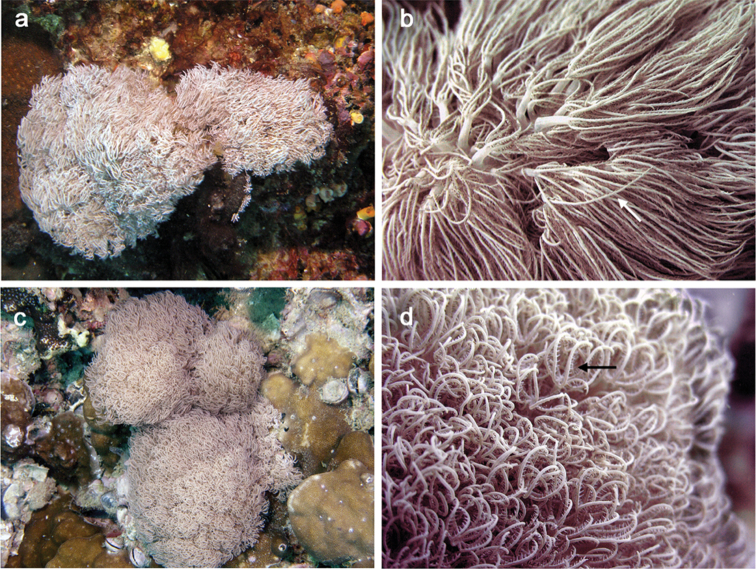
*In situ* images of *Ovabunda andamanensis* sp. n. type material Holotype (PMBC 11860): **a** colony (smallest) **b** close up of polyps. Paratype (PMBC 11862): **c** colony (smallest) **d** close up of polyps.

*Ovabunda* Alderslade, 2001 is a genus of tropical, shallow water zooxanthellate soft corals belonging to the family Xeniidae. They are an abundant component of benthic communities throughout the Red Sea (Reinicke 1997; Benayahu 1990; Benayahu et al. 2002; Halász et al. 2013). To date, *Ovabunda* has been documented from a relatively small geographical range with only three species recorded beyond the Red Sea, occurring in the Seychelles Islands (Janes 2008a) and Madagascar (Halász et. al. 2013), Western Indian Ocean. *Ovabunda* sclerites are unique among xeniids. Unlike the flattened figlet-like sclerites comprised of radially arranged dendritic rods commonly found in many xeniid genera, *Ovabunda* sclerites are round or oval to irregular spheroids comprised of aggregations of minute corpuscular-shaped microscleres (Alderslade 2001). Eleven nominal species previously described as belonging to the genus *Xenia* were reassigned to *Ovabunda* (Alderslade 2001; Janes 2008a; Aharonovich and Benayahu 2011; Halász et al. 2013) primarily based on the type of sclerite they exhibited: *Ovabunda ainex* Reinicke, 1997; *Ovabunda arabica* Reinicke, 1995; *Ovabunda benayahui* Reinicke, 1995; *Ovabunda biseriata* Verseveldt and Cohen, 1971; *Ovabunda crenata* Reinicke, 1997; *Ovabunda faraunensis* Verseveldt and Cohen, 1971; *Ovabunda gohari* Reinicke, 1997; *Ovabunda hamsina* Reinicke, 1997; *Ovabunda impulsatilla* Verseveldt and Cohen, 1971; *Ovabunda macrospiculata* Gohar, 1940; *Ovabunda verseveldti* Benayahu, 1990. An additional species, *Ovabunda andamanensis* sp. n., is described and illustrated below (Fig. 1). Molecular analyses of the *mtMutS*, *COI*, and 28S rDNA genes have shown relatively few genetic differences among species of *Ovabunda* or between *Ovabunda* and some species of *Xenia* (Haverkort-Yeh et al. 2013; McFadden et al. 2014b; A. Halász et al. in review).

Here we describe a new species of *Ovabunda* from recent collections in the Andaman Sea. Additionally, we report the first record of this genus outside of the eastern Indian Ocean and Red Sea.

## Methods

### Collection and Morphological examination

All specimens were collected using SCUBA to a maximum depth of 10 meters. Photographs of living colonies were taken for each specimen. Specimens were fixed in 90% ethyl alcohol immediately after collection. The corals examined in this survey are deposited in the reference collection of the Phuket Marine Biological Center, Phuket, Thailand (PMBC).

Morphological examination of the preserved colonies was performed under a dissecting microscope at 20× power. Polyps were photographed; number of pinnule rows, pinnules along the outermost row and inner row when present for each specimen were recorded. Sclerites were prepared for light microscope examination (Janes 2008b); 20–25 sclerites were selected and measured to the nearest 0.001 mm on a compound microscope fitted with a Filar Micrometer. Scanning electron microscope (SEM) images of sclerites were made from preparations following the method given by Aharonovich and Benayahu (2011). Sclerites were sputter coated with a gold/palladium blend for 75 seconds. They were then examined using a FEI XL30 Environmental Scanning Electron Microscope (ESEM) at 10 kV and a Nova 200 NanoLab Focused Ion Beam (FIB) Microscope at 5 kV, 0.40 nA.

### Molecular phylogenetic analyses

Extraction of DNA from ethanol-preserved tissue samples, PCR amplification, and sequencing of two mitochondrial (*mtMutS*, *COI* + *igr1*) and a nuclear (28S rDNA) gene followed the protocols published in McFadden et al. (2014a). Sequences were aligned to a reference set of published sequences for other xeniid taxa (Haverkort-Yeh et al. 2013; McFadden et al. 2014b) using the L-INS-i method in MAFFT (Katoh et al. 2005). Pairwise genetic distances (Kimura 2-parameter) among specimens were calculated using the DNADist program in PHYLIP v. 3.69 (Felsenstein 2005). MOTHUR v. 1.29 (Schloss et al. 2009) was used to cluster sequences into molecular taxonomic units (MOTUs) based on an average neighbour distance threshold of 0.3%, a value that has been shown previously to yield a high concordance between molecular and morphological identifications in other octocoral taxa including xeniids (McFadden et al. 2014b). Modeltest 3.0 (Posada and Crandall 1998) was used to select appropriate models of evolution for maximum likelihood analyses that were run for 100 bootstrap replicates using GARLI 2.0 (Zwickl 2006). The 28S rDNA and mitochondrial gene (*mtMutS* + *igr1* + *COI*) datasets were analyzed separately, and in a combined analysis with different models of evolution applied to separate data partitions (mt genes: HKY+I+G; 28S: GTR+I+G). Bayesian analyses were run using MrBayes v. 3.2.1 (Ronquist et al. 2012) with the same data partitions. Analyses were run for 2,000,000 generations (until standard deviation of split partitions < 0.005) with a burn-in of 25% and default Metropolis coupling parameters (i.e., 2 runs, 4 chains (3 heated), sample frequency = 500 generations).

## Results

### Systematic section
Order Alcyonacea Lamouroux, 1812
Family Xeniidae Ehrenberg, 1828 Genus *Ovabunda* Alderslade, 2001: 49–52

#### Ovabunda andamanensis sp. n.

Taxon classificationAnimaliaAlcyonaceaXeniidae

http://zoobank.org/F5E7EEC2-A203-491D-8C2C-5DE90C1F2404

Figs 1 [Fig F2] [Fig F3] [Fig F4] –5

##### Material.

**Holotype:** PMBC 11860, Koh Doc Mai, Thailand, 07°47.76'N, 98°32.09'E, depth 8 meters, 26 September 2007 (1 colony), M. P. Janes collector. **Paratypes:** PMBC 11861, Koh Phi Phi, Hin Bida, Thailand, 07°39.20'N, 98°45.83'E, depth 10 meters, 28 September 2007 (1 colony), M. P. Janes collector. PMBC 11862, same data as the holotype.

##### Description.

**Figure 2. F2:**
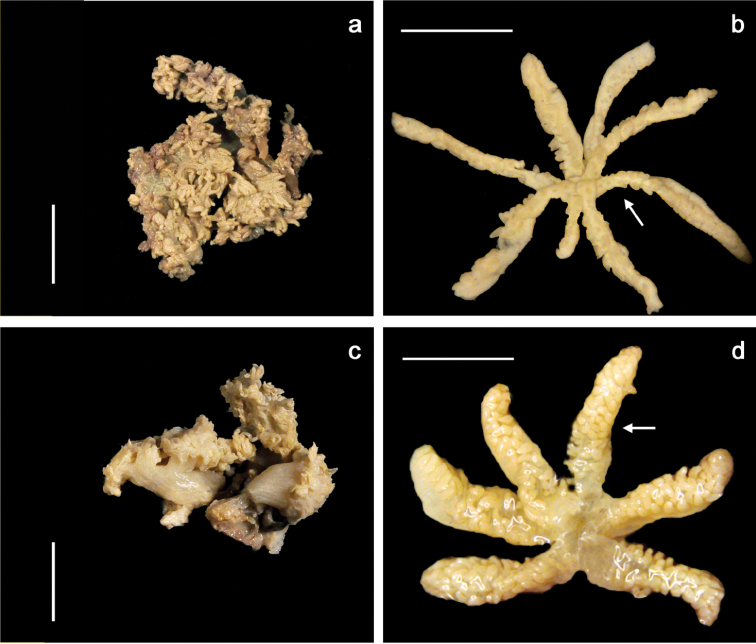
Preserved specimens of *Ovabunda andamanensis* sp. n. Holotype (PMBC 11860): **a** colony **b** polyp. Paratype (PMBC 11862): **c** colony **d** polyp. Scale bars: **a** and **c** 10 mm; **b** and **d** 4 mm.

The holotype is comprised of multiple short, branched stalks sharing a common base attached to coral rock. In life, the holotype (Fig. 1a) is thickly beset with monomorphic polyps which were observed to be non-pulsatile. The tentacles are cylindrical, slender and up to 2.0 cm long (Fig. 1b). In a preserved state, the holotype consists of multiple stalks; two of the stalks are divided into two short branches (Fig. 2a). The stalks measure up to 8.0 mm tall and 5.0 mm wide at the base. A slightly convex capitulum is present at the distal end of the stalks from which moderately dense aggregations of polyps are growing. The polyp bodies are cylindrical, shrunken and measuring from their attachment at the capitulum to the base of the tentacle they are 1.5 mm long by 0.5 mm wide. Tentacles are slender, measuring up to 8.0 mm long by 0.2 mm wide with a blunt tip. There is one row of pinnules present on either side of the tentacle (Fig. 2b at arrow) with 17 to 19 pinnules in a row. The pinnules are barrel-like with a rounded tip and slight taper at the end. They measure 0.2 to 0.3 mm long by 0.1 mm wide. There is an open space on the tentacles between adjacent pinnules measuring 0.1 to 0.15 mm wide, nearly equal to their width. Zooxanthellate.

**Figure 3. F3:**
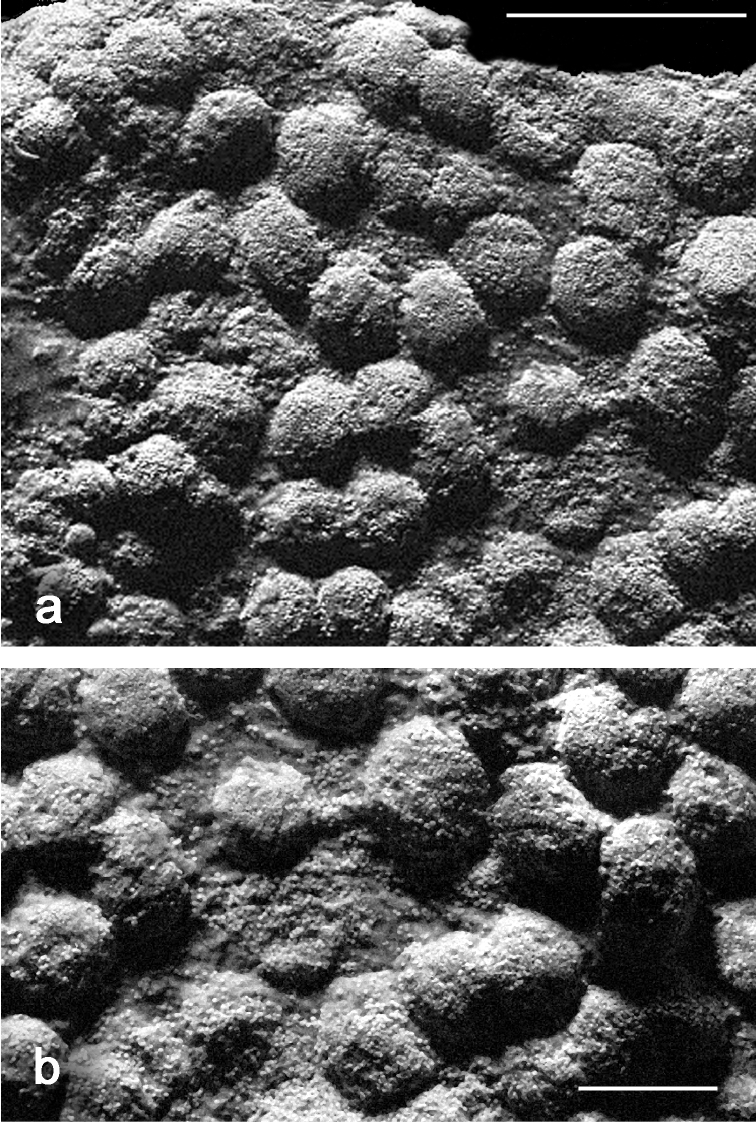
*Ovabunda andamanensis* sp. n. Holotype (PMBC 11860) sclerites: **a–b** uncoated ESEM wet mount images of tentacle sclerites *in situ*. Scale bars: **a** 0.04 mm; **b** 0.015 mm.

**Figure 4. F4:**
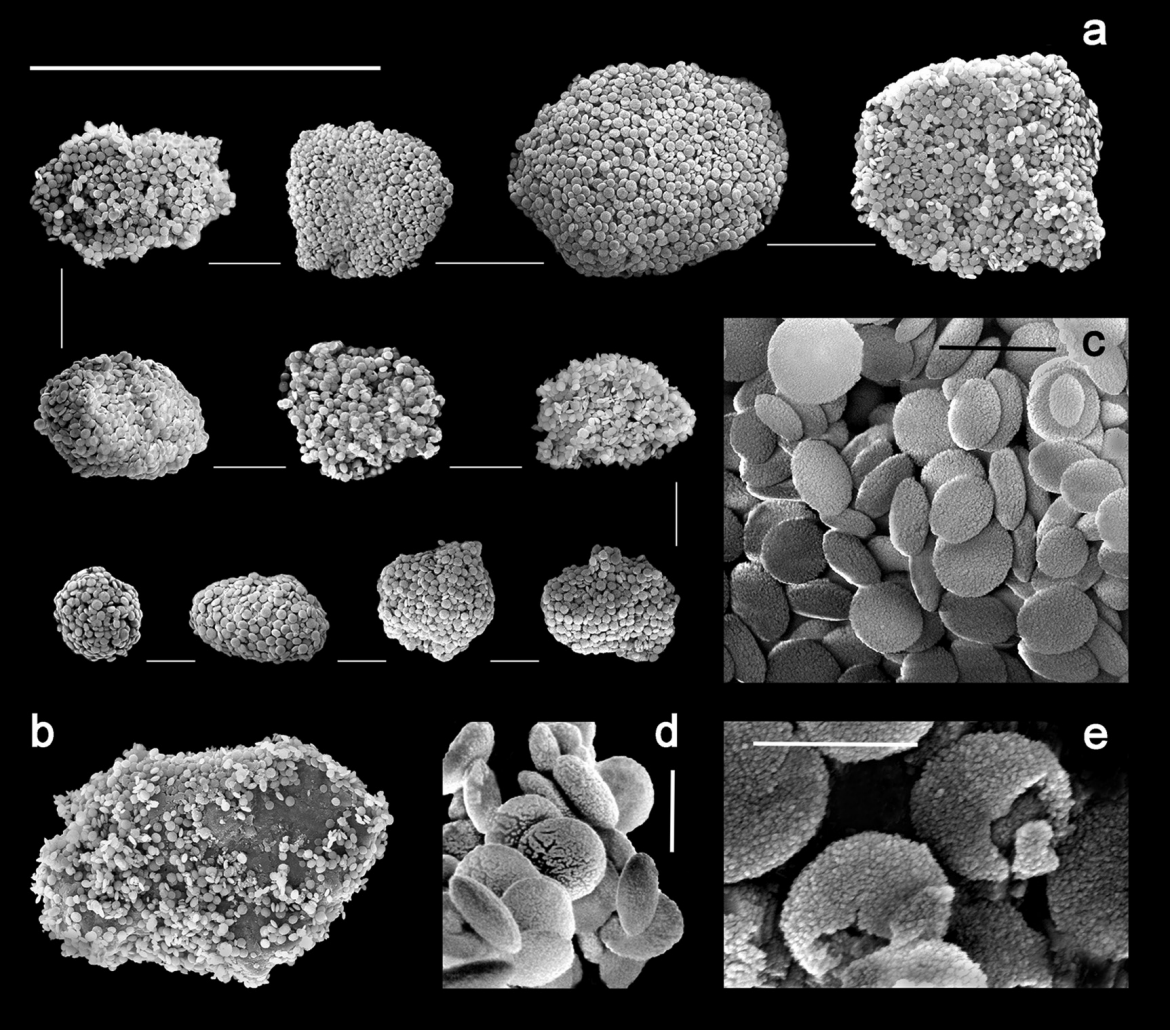
*Ovabunda andamanensis* sp. n. Holotype (PMBC 11860) SEM image of coated sclerites: **a** whole sclerite most common form **b** whole sclerite least common form **c** microscleres from a disintegrated sclerite **d** surface ultrastructure of loose microscleres **e** fractured microscleres. Scale bars: **a–b** 0.020 mm; **c** 0.001 mm; **d–e** 0.0005mm.

**Figure 5.**
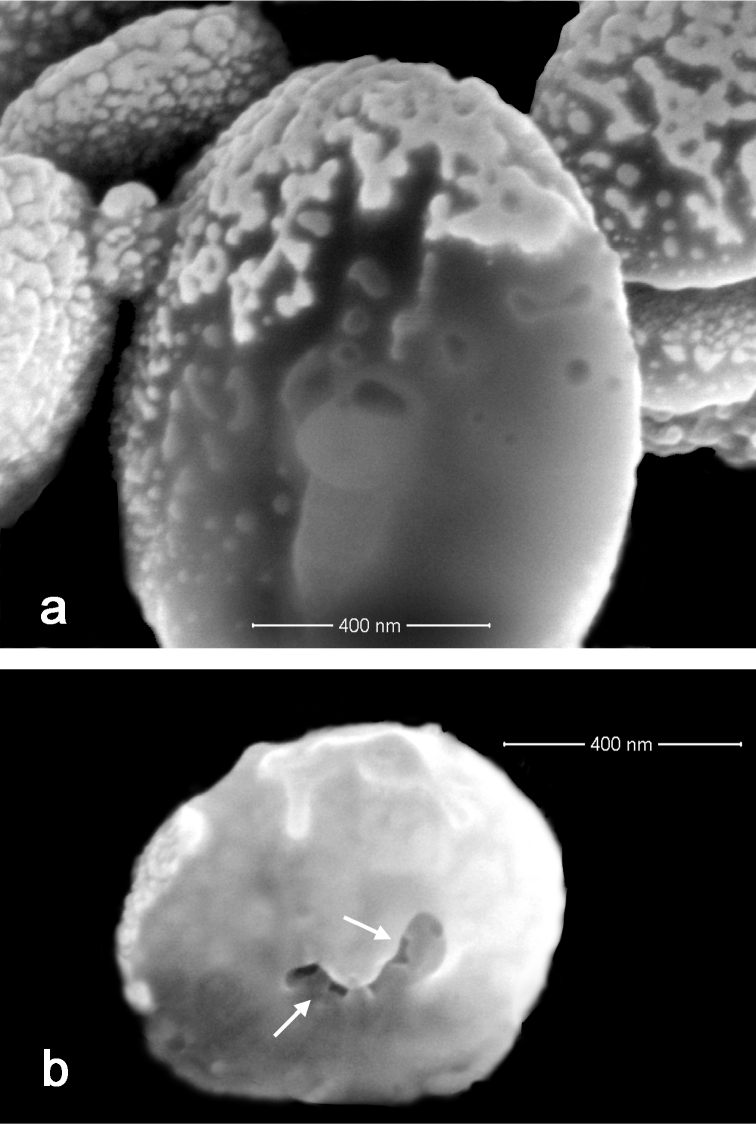
*Ovabunda andamanensis* sp. n. Holotype (PMBC 11860) sclerites: **a** FIB image of the surface ultrastructure of microscleres **b** FIB image showing interior cavities of a microsclere.

Sclerites are present in all parts of the holotype. They are moderately dense in the polyps (Fig. 3a–b) and fewer are found in the basal portion of the stalk. All of the sclerites are round to slightly oval or irregularly shaped spheroids (Fig. 4a). They measure from 0.010 to 0.018 mm in diameter on average with a few sclerites as large as 0.020 mm in maximal diameter and the smallest recorded at 0.005 mm in diameter. The sclerites are comprised of numerous, minute circular to egg-shaped corpuscular microscleres that quickly disassociate when extracted from the coral tissue with sodium hypochlorite. Rarely, a second form of sclerite can be found in the polyp tissue, which contains a solid calcite core coated with microscleres (Fig. 4b). The microscleres measure from 0.00035 to 0.00060 mm in diameter. SEM imaging revealed that they have a fine, granular surface ultrastructure (Fig. 4c) when viewed under moderately high magnification (×40,000). The ultrastructure of the surface is comprised of a series of ridges, furrows, and coarse nodules (Fig. 4d) when examined at 65,000 power. Most microscleres are intact but occasionally fractured ones are found (Fig. 4e). Broken microscleres reveal the presence of a cavity or cavities within. Further evidence of these can be seen in FIB imaging where the microsclere has been sliced longitudinally, demonstrating the depth of the furrows (Fig. 5a) and size of cavities (Fig. 5b) when magnified at 200,000 power.

##### Color.

The preserved specimens are cream colored with whitish tentacles and light tan pinnules. Living colonies exhibit tan colored stalks; white polyp bodies and tentacles, and pinkish pinnules (Fig. 1a–d).

##### Etymology.

The name is derived from the collection location, the Andaman Sea.

##### Distribution and ecology.

**Figure 6.**
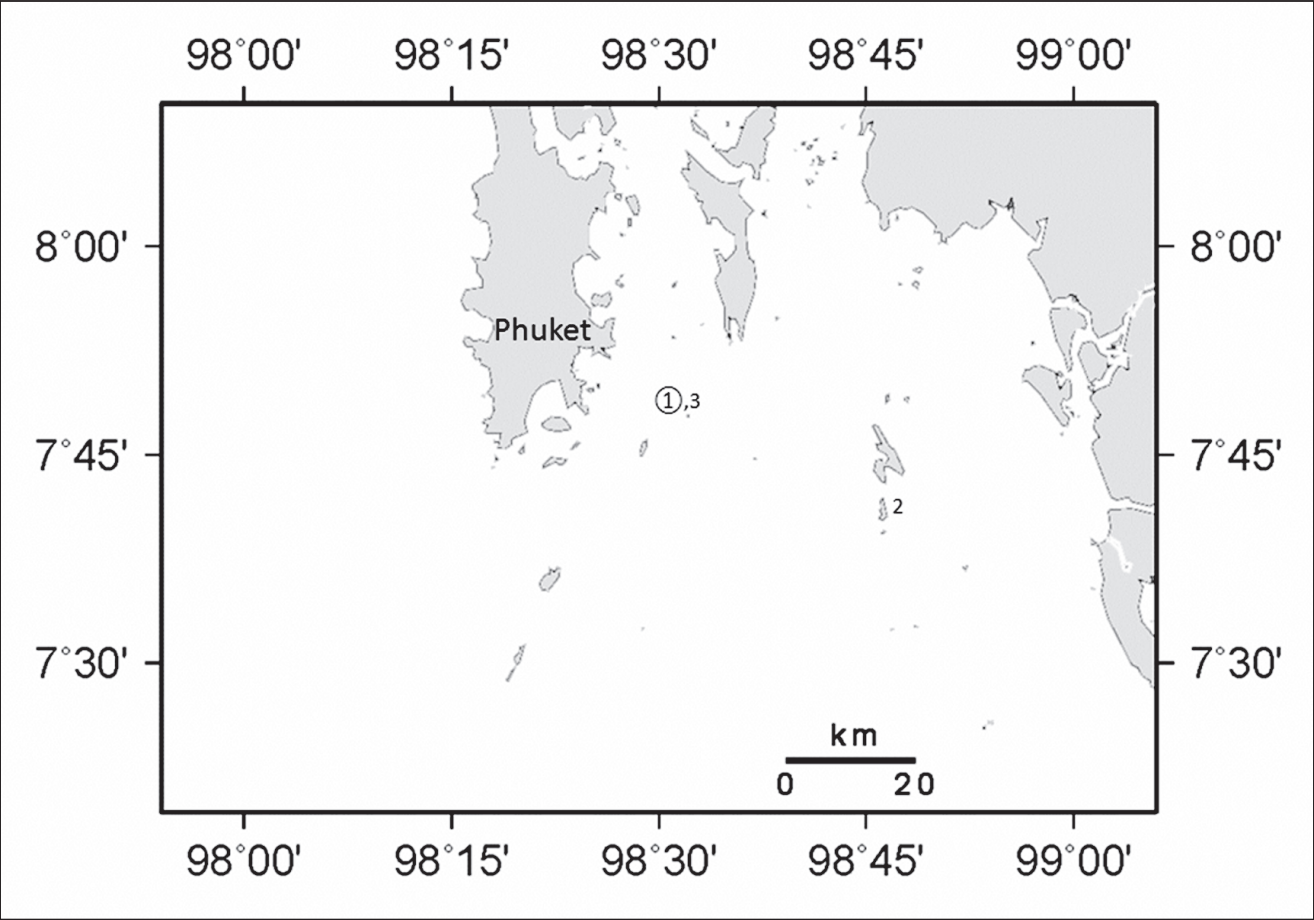
Distribution of *Ovabunda andamanensis* sp. n. in the Andaman Sea: **1** PMBC 11860 **2** PMBC 11861 **3** PMBC 11862. Circled number denotes holotype.

This species was collected from Koh Doc Mai (Fig. 6) and Koh Phi Phi, Hin Bida located along the eastern coast of Phuket Island, Thailand. Colonies occur in low abundance, spaced 1-2 meters apart on small ledges of vertical walls growing among sea fans, corallimorpharians, and *Dendronephthya* sp. soft corals. *Ovabunda andamanensis* sp. n. has also been observed *in situ* in the Mergui Archipelago, Myanmar (T. Chanmethakul, personal observation).

##### Variability.

Both the holotype (PMBC 11860) and one paratype (PMBC 11861) were very similar in size (up to 7.0 cm in life), sclerite dimensions, exhibited non-pulsatile polyps, pinnule rows and number of pinnules per row. The other paratype (PMBC 11862) was smaller in life (Figs 1c, 2c), measuring up to 5.0 cm tall and exhibited tentacles that curved inward (Fig. 1d). It differed from the holotype in having shorter tentacles and two rows of 13–14 pinnules in each row (Fig. 2d) instead of one row with 17–19 pinnules.

##### Remarks.

In comparing the morphology of *Ovabunda andamanensis* sp. n. to nominal *Ovabunda* species colony size, sclerite size and shape, and pinnule arrangement were examined, with the later considered a more variable diagnostic character. Among species of *Ovabunda* described as having one or two rows of pinnules, *O. arabica, O. biseriata, O. gohari, O. faraunensis* and *Ovabunda verseveldti* most closely resemble *Ovabunda andamanensis* sp. n. *Ovabunda gohari* and *Ovabunda verseveldti* are similar to the holotype in having primarily one row of pinnules although occasionally two rows are present in polyps of *Ovabunda gohari*. The overall range of sclerite sizes is larger in *Ovabunda gohari*, 0.033–0.063 mm, compared to the 0.010–0.018 mm in *Ovabunda andamanensis* sp. n., there are 18–22 pinnules in a row compared to 17–19 pinnules in *Ovabunda andamanensis* sp. n., and *Ovabunda gohari* has pinnules spaced at 2–3 times the pinnule width along the tentacles whereas in *Ovabunda andamanensis* sp. n. pinnules are more closely set. According to Reinicke (1997: 31) *Ovabunda gohari* sclerites are “… packed between polyp bases and in the syndete’s wall between mesenteries, giving a longitudinal whitish banding”; this feature was not observed in the Thailand material. *Ovabunda verseveldti* contains much larger sclerites (up to 0.030 × 0.049 mm) than were found in *Ovabunda andamanensis* sp. n. and the pinnules are “densely set in each row, almost touching each other” (Halász et al. 2013) compared to the wider pinnule spacing in the Thailand specimens.

The pinnule number in *Ovabunda faraunensis* agrees well with the 17–19 pinnules observed in the holotype of *Ovabunda andamanensis* sp. n., however *Ovabunda faraunensis* has larger sclerites (Reinicke 1997: 35). The sclerite sizes in *Ovabunda arabica* 0.028–0.036 mm (Halász et al. 2013), *Ovabunda biseriata* 0.018–0.035 mm (Verseveldt and Cohen 1971: 60), 0.029–0.063 mm (Reinicke 1997: 33), and *Ovabunda faraunensis* 0.028–0.044 mm (Halász et al. 2013) are all notably larger than the average range of 0.010-0.018 mm observed in *Ovabunda andamanensis* sp. n.

Among species of *Xenia*, *Xenia puerto-galerae* Roxas, 1933 most closely resembles *Ovabunda andamanensis* sp. n. The holotype is described by Roxas (1933) as branched, measuring 20.0 mm tall and 8.0 mm in diameter with polyps comprised of thick tentacles that are proportionately small and “two rows of slender pointed pinnules, fifteen to seventeen in a row”. The tentacles are 8.0 mm long by 1.0 mm wide at the base and pinnules measure 0.7 to 0.8 mm long by 0.2 to 0.3 mm wide. However, in the colony with two rows (PMBC 11862) in *Ovabunda andamanensis* sp. n., the stalks are smaller, 8.0 mm by 5.0 mm, the tentacles are narrower, 8.0 mm by 0.2 mm, the pinnules are smaller, 0.2 to 0.3 mm by 0.1 mm, and there are fewer pinnules (13 to 14). Most notable are the sclerites, which are described as “thin, oval discs 0.018 mm long and 0.018 to 0.0124 mm wide” in *Xenia puerto-galerae* compared to the 0.010 to 0.018 mm in diameter sphere shaped sclerites observed in *Ovabunda andamanensis* sp. n. Unfortunately, the location of the holotype of *Xenia puerto-galerae* remains unknown so a direct SEM comparison of the sclerites could not be performed.

### Molecular analysis

**Figure 7.**
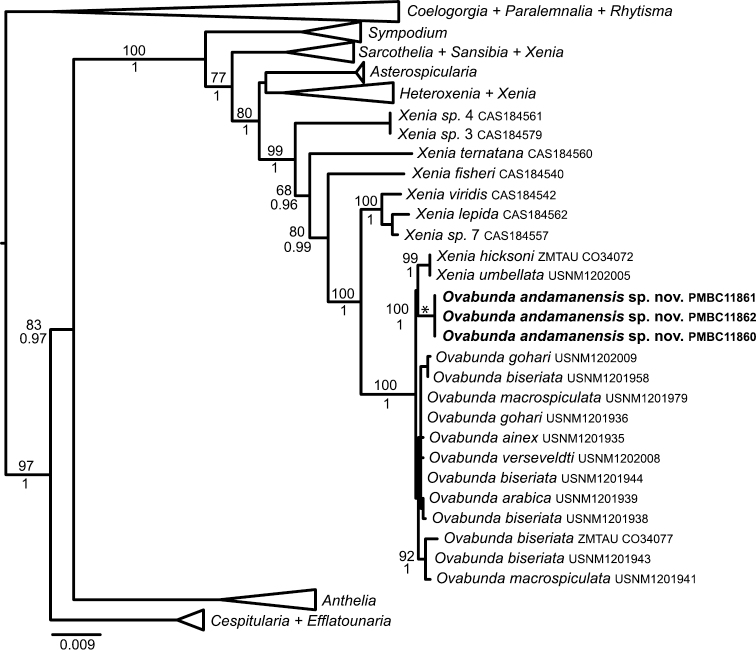
Maximum likelihood reconstruction of family Xeniidae based on a partitioned analysis of *mtMutS*, *COI* and 28S rDNA sequences (2255 bp). Numbers above nodes are bootstrap percentages (100 replicates) from ML analyses; numbers below nodes are Bayesian posterior probabilities. Some clades have been collapsed to triangles to facilitate readability.

All three *Ovabunda* specimens in this collection had identical genotypes at *mtMutS*, *COI* + *igr1* and 28S rDNA. Phylogenetic analyses placed them in a well-supported clade with all other species of *Ovabunda* as well as several species of *Xenia* from the Red Sea (Fig. 7). Within that clade, only four MOTUs were distinguished by an average genetic distance of 0.3% or greater. The three Thai specimens belonged to a MOTU that was separated from all other species by 0.5%. The two species of *Xenia* (*Xenia umbellata*, *Xenia hicksoni*) found within the clade belonged to a MOTU that was separated from Red Sea *Ovabunda* species by 0.4%. All Red Sea *Ovabunda* species belonged to just two MOTUs that were separated by 0.3%: the group of [USNM1201941, USNM1201943 and ZMTAUCO34077] and all others. Based on its sclerite size, colony size and form, pinnule arrangement, and unique phylogenetic position, we hereby designate a new species *Ovabunda andamanensis* sp. n. for our material.

## Discussion

**Figure 8.**
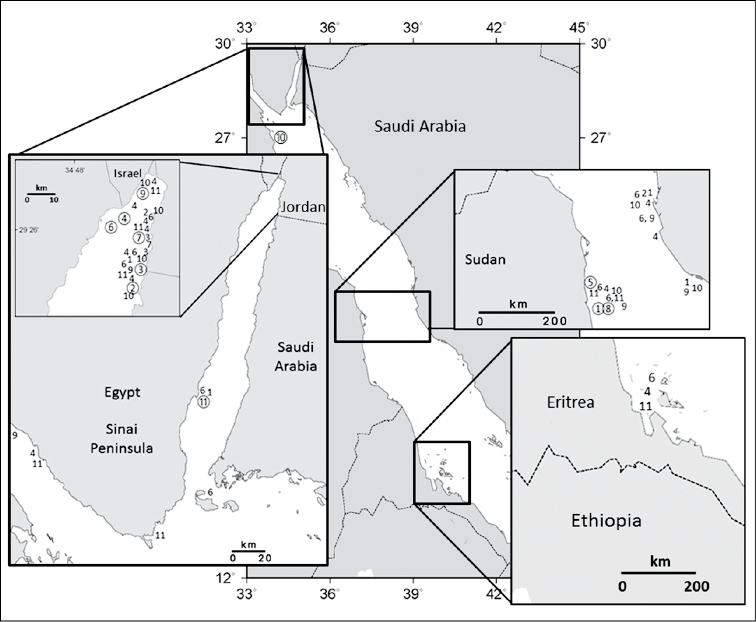
Distribution of *Ovabunda* species in the Red Sea: **1** *Ovabunda ainex* **2** *Ovabunda arabica* **3** *Ovabunda benayahui* **4** *Ovabunda biseriata*; **5** *Ovabunda crenata*; **6** *Ovabunda faraunensis*; **7** *Ovabunda gohari* **8** *Ovabunda hamsina* **9** *Ovabunda impulsatilla* **10** *Ovabunda macrospiculata* **11** *Ovabunda verseveldti*. Circled number denotes holotype.

**Figure 9.**
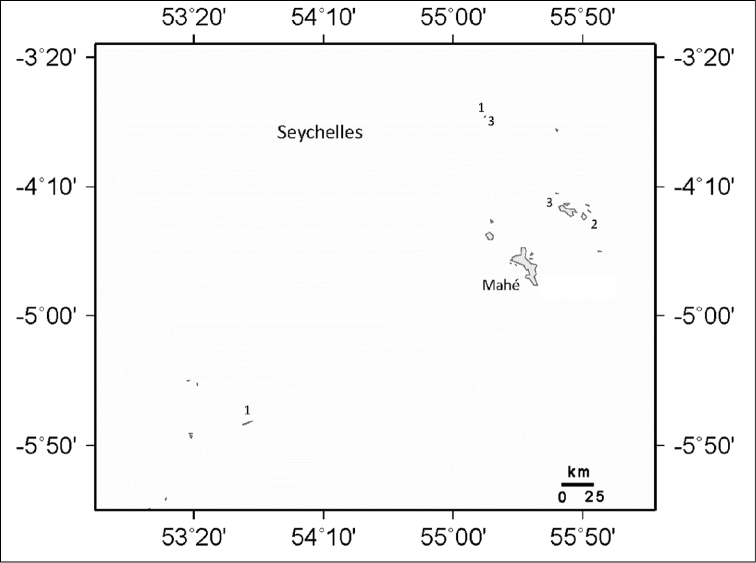
Distribution of *Ovabunda* species in the Seychelles Islands: **1** *Ovabunda impulsatilla* **2** *Ovabunda benayahui* **3** *Ovabunda hamsina*.

Octocorals are thought to exhibit their greatest species richness in the Indo-Malayan region consisting of Indonesia, the Philippines and New Guinea (Ekman 1953; Hoeksema and Ofwegen 2004; Ofwegen 2005; Wood and Dipper 2008; Hoeksema 2009), an area also known for its scleractinian diversity (Baird et al. 2009; Veron et al. 2009). *Ovabunda* is mainly found in the Red Sea, where the highest number of species (11) occurs (Fig. 8). Benayahu et al. (2002) note only three species from the southern Red Sea, *Ovabunda biseriata*, *Ovabunda faraunensis*, and *Ovabunda verseveldti*. A higher number of *Ovabunda* species (10) are recorded from the central Red Sea including the holotypes of *Ovabunda ainex*, *Ovabunda crenata*, and *Ovabunda hamsina*. This is likely due to extensive collection efforts in Sudan (Reinicke 1995, 1997) and Saudi Arabia (Haverkort-Yeh et al. 2013). Similarly, nine species have been identified from the northern Red Sea in the Gulf of Suez and the Gulf of Aqaba. Extensive octocoral research in Eilat, Israel (Benayahu and Loya 1977, 1981, 1985; Benayahu 1990) accounts for the numerous records at the northern most end of the Gulf of Aqaba. Janes (2008a) recorded three species from the Seychelles Islands (Fig. 9). There are additional reports of specimens collected from Madagascar (Reinicke 1997; Halász et al. 2013) and Reunion Island (Benayahu and Ofwegen 2012) in the Western Indian Ocean.

This new record from Thailand increases the know distribution of *Ovabunda* in the Indian Ocean approximately 5000 km eastward. Both the holotype and one paratype of *Ovabunda andamanensis* sp. n. were collected from Koh Doc Mai (Fig. 6) located along the eastern coast of Phuket Island. An additional paratype was collected from Koh Phi Phi, Hin Bida located at the southern end of the Gulf of Thailand. Xeniids have been recorded previously from the Andaman Sea (Chanmethakul et al. 2010) so it is likely that this or other species of *Ovabunda* will be found elsewhere in the Andaman Sea or eastern Indian Ocean if a thorough octocoral survey is carried out. Specimens of *Ovabunda andamanensis* sp. n. were all collected from horizontal shelves on vertical walls, and it is perhaps on horizontal surfaces such as this where *Ovabunda* species might occur.

The gross morphology, sclerite dimensions, and molecular results all support the description of *Ovabunda andamanensis* sp. n. as a new species. The range of intraspecific variability among xeniid species is poorly known. Living colonies of *Ovabunda andamanensis* sp. n. were observed to be smaller and less numerous than *Ovabunda* colonies found on the coral reefs in the northern Gulf of Aqaba (M. Janes, personal observation) and the largest sclerites found in *Ovabunda andamanensis* sp. n. had a maximal diameter of 0.020 mm, notably smaller than the 0.035–0.040 mm maximal diameter recorded by Halász et al. (2013: 36) in Red Sea species; whether this is a result of environmental factors such as light or water flow (Reinicke 1995) or is typical for colonies of *Ovabunda andamanensis* sp. n. remains unknown. The solid sclerite form (Fig. 4b) coated with microscleres that was rare in the Thai material is similar to sclerites recorded from *Ovabunda impulsatilla* collected in the Seychelles (Janes 2008a: 612). While the smaller maximal diameter of sclerites from *Ovabunda andamanensis* sp. n. are considered a taxonomic character of this species, the occasional hollow cavities in the microscleres are not; primarily due to limited sampling of the microscleres.

The results of our study suggest both the holotype and two paratypes of *Ovabunda andamanensis* sp. n. are the same species which can exhibit one or two rows of pinnules. Previous authors have suggested the number of rows of pinnules can be variable within a species and is therefore not a highly reliable taxonomic character. Early on both Thomson and Henderson (1906: 410) and Kükenthal (1913: 6) recognized the variability in pinnule size and their arrangement. Later, Gohar (1940: 79) noted that “The number of rows increases with age…” and cited *Heteroxenia fuscescens* juveniles with two rows of pinnules where the adult colonies had up to five. Further, in their revision of *Ovabunda* Halász et al. (2013) mentioned the difficulty in identifying irregular rows of pinnules in the species *Ovabunda crenata* and *Ovabunda hamsina*. All three Thailand samples were nearly identical with regards to gross morphology, sclerite shape and size, pinnule spacing, lack of pulsation, and molecular genotype. *Ovabunda andamanensis* sp. n. differs from other *Ovabunda* species by having the following unique combination of characters: sclerites measuring from 0.010 to 0.018 mm in diameter on average, small colonies with slender tentacles up to 2.0 cm long in life, exhibits one or two rows of pinnules, and distinguished from all other *Ovabunda* species by a genetic distance of 0.5%.

## Supplementary Material

XML Treatment for Ovabunda andamanensis

## Supplementary material

Supplementary material 1 Supplement S1. doi: 10.3897/zookeys.431.7751.app1 Data type: specimens data Explanation note: Specimens and sequences included in molecular phylogenetic analysis of xeniids. This dataset is made available under the Open Database License (http://opendatacommons.org/licenses/odbl/1.0/). The Open Database License (ODbL) is a license agreement intended to allow users to freely share, modify, and use this Dataset while maintaining this same freedom for others, provided that the original source and author(s) are credited.Catherine S. McFadden

## References

[B1] Aharonovich D Benayahu Y (2011) Microstructure of octocoral sclerites for diagnosis of taxonomic features. Marine Biodiversity 42(2): 173–177. doi: 10.1007/s12526-011-0102-3

[B2] Alderslade P (2000) Four new genera of soft corals (Coelenterata: Octocorallia), with notes on the classification of some established taxa. Zoologische Mededelingen 74(16): 237–249

[B3] Alderslade P (2001) Six new genera and six new species of soft coral, and some proposed familial and subfamilial changes within the Alcyonacea (Coelenterata: Octocorallia). Bulletin of the Biological Society of Washington 10: 15–65

[B4] Baird AH Guest JR Willis BL (2009) Systematic and biogeographical patterns in the reproductive biology of scleractinian corals. Annual Review of Ecology, Evolution, and Systematics 40: 551–571. doi: 10.1146/annurev.ecolsys.110308.120220

[B5] Benayahu Y Loya Y (1977) Space partitioning by stony corals soft corals and benthic algae on the coral reefs of the northern Gulf of Eilat (Red Sea). Helgolander wissenschaftliche Meeresuntersuchungen 30: 362–382. doi: 10.1007/BF02207848

[B6] Benayahu Y Loya Y (1981) Competition for space among coral reef sessile organisms at Eilat, Red Sea. Bulletin of Marine Science 31: 514–522

[B7] Benayahu Y Loya Y (1985) Settlement and recruitment of a soft coral: Why is Xenia macrospiculata a successful colonizer? Bulletin of Marine Science 36: 177–188.

[B8] Benayahu Y (1990) Xeniidae (Cnidaria: Octocorallia) from the Red Sea, with the description of a new species. Zoologische Mededelingen 64: 113–120

[B9] Benayahu Y Yosief T Schleyer HM (2002) Soft corals (Octocorallia, Alcyonacea) of the Southern Red Sea. Israel Journal of Zoology 48: 273–283. doi: 10.1560/HYC7-TUTH-EV77-BEUQ

[B10] Benayahu Y Ofwegen LP van (2012) Octocorals (Cnidaria, Anthozoa) from Reunion, with a description of two new species of the genus *Sinularia* May, 1898 and notes on the occurrence of other species. Zoosystema 34: 673–699. doi: 10.5252/z2012n4a2

[B11] Chanmethakul T Chansang, H Watanasit S (2010) Soft coral (Cnidaria: Alcyonacea) distribution patterns in Thai waters. Zoological Studies 49: 72–84

[B12] Ehrenberg CG (1834) Beitrage zur physiologischen Kenntniss der Corallenthiere im allgemeinen, und besonders des rothen Meeres, Nebst einem Versuche zur physiologischen Systematik derselben. Abhandlungen der Koniglichen Akademie der Wissenschaften zu Berlin, 225–287.

[B13] Ekman S (1953) Zoogeography of the Sea. Sidgwick and Jackson, London, 440 pp

[B14] Fabricius K Alderslade P (2001) Soft Corals and Sea Fans: A Comprehensive Guide to the Tropical Shallow-Water Genera of the Central-West Pacific, Indian Ocean and the Red Sea. Australian Institute of Marine Science, Townsville, 264 pp

[B15] Felsenstein J (2005) PHYLIP (Phylogeny Inference Package) version 3.6. Distributed by the author. Department of Genome Sciences, University of Washington, Seattle.

[B16] Gohar HAF (1940) Studies on the Xeniidae of the Red Sea Their Ecology, Physiology, Taxonomy and Phylogeny. Publications of the Marine Biological Station Gharadaqa (Red Sea) 2: 25–118

[B17] Halász A McFadden CS Aharonovich D Toonen R Benayahu Y (2013) A revision of the octocoral genus *Ovabunda* (Alderslade, 2001) (Anthozoa, Octocorallia, Xeniidae). ZooKeys 373: 1–41. doi: 10.3897/zookeys.373.6511 24493958 10.3897/zookeys.373.6511 PMC3909805

[B18] Haverkort-Yeh RD McFadden CS Benayahu Y Berumen M Halász A Toonen R (2013) A taxonomic survey of Saudi Arabian Red Sea octocorals (Cnidaria: Alcyonacea). Marine Biodiversity 43: 279–291. doi: 10.1007/s12526-013-0157-4

[B19] Hoeksema BW Ofwegen LP van (2004) Reef corals of Indonesia and SE Asia: a generic overview. World Biodiversity Database CD-ROM Series ETI, Amsterdam.

[B20] Hoeksema BW (2009) West-East variation in the Indonesian reef coral fauna: lines of division or zones of transition? World Ocean Conference, Manado, Indonesia, May 11–15, 2009.

[B21] Janes M (2008a) A study of the Xeniidae (Octocorallia, Alcyonacea) collected on the “Tyro” expedition to the Seychelles with a description of a new genus and species. Zoologische Mededelingen 82: 599–626

[B22] Janes M (2008b) Laboratory Methods for the identification of soft corals (Octocorallia: Alcyonacea). In: Leewis RJ Janse M (Eds) Advances in Coral Husbandry in Public Aquariums.Public Aquarium Husbandry Series, vol. 2. 1st International Symposium of Coral Husbandry in Public Aquaria, Arnhem (The Netherlands), April, 2007. Burgers’ Zoo, 413–426

[B23] Katoh K Kuma K Toh H Miyata T (2005) MAFFT version 5: improvement in accuracy of multiple sequence alignment. Nucleic Acids Research 33: 511–513. doi: 10.1093/nar/gki198 15661851 10.1093/nar/gki198 PMC548345

[B24] Kükenthal W (1913) Alcyonaria des Rothen Meers. Expeditionen S.M. Schiff “Pola” in das Rote Meer 1895/96–1897/98. Zoologische Ergebnisse 29: 1–32

[B25] Lamouroux JVF (1812) Memoires sur la montee et sur une nouvelle classification des polypiers coralligenes non entierement pierreux. Nouveau Bulletin Society Philomath, Paris, 181–188.

[B26] McFadden CS Brown AS Brayton C Hunt CB Ofwegen LP van (2014a) Application of DNA barcoding in biodiversity studies of shallow-water octocorals: molecular proxies agree with morphological estimates of species richness in Palau. Coral Reefs 33: 275–286. doi: 10.1007/s00338-013-1123-0

[B27] McFadden CS Reynolds AM Janes MP (2014b) DNA barcoding of xeniid soft corals (Octocorallia: Alcyonacea: Xeniidae) from Indonesia: species richness and phylogenetic relationships. Systematics & Biodiversity 12: 247–257. doi: 10.1080/14772000.2014.902866

[B28] Ofwegen LP van (2005) A new genus of nephtheid soft corals (Octocorallia: Alcyonacea: Nephtheidae from the Indo-Pacific. Zoologische Mededelingen 79: 1–236

[B29] Posada D Crandall KA (1998) Modeltest: testing the model of DNA substitution. Bioinformatics 14: 817–818. doi: 10.1093/bioinformatics/14.9.817 9918953 10.1093/bioinformatics/14.9.817

[B30] Reinicke GB (1995) Xeniidae des Roten Meeres (Octocorallia, Alcyonacea) Beitrage zur Systematik und Okologie. Essener Okologische Schriften 6: 1–168

[B31] Reinicke GB (1997) Xeniidae (Coelenterata: Octocorallia) of the Red Sea, with descriptions of six new species of Xenia. Fauna Saudi Arabia 16: 5–62

[B32] Ronquist FM Teslenko M van der Mark P Ayres DL Darling A Höhna S Larget B Liu L Suchard MA Huelsenbeck JP (2012) MrBayes 3.2: Efficient Bayesian phylogenetic inference and model choice across a large model space. Systematic Biology 61: 539–542. doi: 10.1093/sysbio/sys029 22357727 10.1093/sysbio/sys029 PMC3329765

[B33] Schloss PD Westcott SL Ryabin T Hall JR Hartmann M Hollister EB Lesniewski RA Oakley BB Parks DH Robinson CJ Sahl JW Stres B Thallinger GG Van Horn DJ Weber CF (2009) Introducing mothur: Open-source, platform-independent, community-supported software for describing and comparing microbial communities. Applied and Environmental Microbiology 75: 7537–7541. doi: 10.1128/AEM.01541-09 19801464 10.1128/AEM.01541-09 PMC2786419

[B34] Thomson JA Henderson WD (1906) The marine fauna of Zanzibar and British East Africa, from collections made by Cyril Crossland in the years 1901 and 1902. Alcyonaria. Proceedings of the Zoological Society of London 1: 393–443

[B35] Veron JEN Devantier LM Turak E Green AL Kininmonth S Stafford-Smith M Peterson N (2009) Delineating the coral triangle. Galaxea 11: 91–100. doi: 10.3755/galaxea.11.91

[B36] Verseveldt J (1969) A new species of the genus *Anthelia* (Octocorallia: Alcyonacea) from the Gulf of Aqaba (Red Sea). Israel Journal of Zoology 18: 325–327

[B37] Verseveldt J Cohen J (1971) Some new species of Octocorallia from the Gulf of Eilat (Red Sea). Israel Journal of Zoology 20: 53–67

[B38] Wood E Dipper F (2008) What is the future for extensive areas of reef impacted by fish blasting and coral bleaching and now dominated by soft corals? A case study from Malaysia. In: Riegl BM Dodge RE (Eds) Proceedings of the 11^th^ International Coral Reef Symposium, Fort Lauderdale (USA), July 2008. Nova Southeastern University, USA, 1, 410–414

[B39] Zwickl DJ (2006) Genetic algorithm approaches for the phylogenetic analysis of large biological sequence datasets under the maximum likelihood criterion. PhD thesis, University of Texas, Austin, Texas.

