# Supplementary material for: A new species of Ovabunda (Octocorallia, Xeniidae) from the Andaman Sea, Thailand with notes on the biogeography of this genus
Source: Zookeys. 2014 Aug 5;(431):1–17. doi: 10.3897/zookeys.431.7751 (PMC4141170; doi:10.3897/zookeys.431.7751)
Supplement: Supplementary material 1 — Supplement S1. [file zookeys-431-001-s001.doc]

**Supplement S1**. Specimens and sequences included in molecular phylogenetic analysis of xeniids. Specimens from other locations indicated in boldface. CASIZ: California Academy of Sciences; NTM: Museum and Art Gallery of the Northern Territory; PMBC: Phuket Marine Biological Center, Thailand; RMNH: Naturalis Biodiversity Center (formerly Rijksmuseum van Natuurlijke Historie), Leiden; USNM: National Museum of Natural History, Washington, D.C.; ZMTAU: Zoological Museum, Tel Aviv University. CSM: Laboratory of C.S. McFadden. NA: sequence not available. xxxxxxx = GenBank accession numbers still to be added.

|  |  |  | | |
| --- | --- | --- | --- | --- |
| **Genus,**  **Species** | **Voucher** | ***COI*** | ***mtMutS*** | ***28S rDNA*** |
| ***Anthelia*** |  |  |  |  |
| *A. glauca* | ZMTAU CO 34183 | GQ342460 | JX203812 | JX203753 |
| *A. philippinense* | CASIZ 184550 | KJ511373 | KJ511334 | KJ511294 |
| *Anthelia* sp. | CASIZ 184543 | KJ511371 | KJ511332 | KJ511292 |
| *Anthelia* sp. | USNM1201961 | KC864943 | KC864878 | xxxxxxxx |
| ***Asterospicularia*** |  |  |  |  |
| *A. laurae* | OCDN8971L | xxxxxxxx | xxxxxxxx | xxxxxxxx |
| *A. randalli* | RMNH Coel. 41520 | KF955018 | KF915555 | KF915315 |
| ***Cespitularia*** |  |  |  |  |
| *C. simplex* | CASIZ 184569 | KJ511377 | KJ511337 | KJ511298 |
| ***Efflatounaria*** |  |  |  |  |
| *Efflatounaria* sp. | NTM C012311 | NA | DQ302838 | NA |
| ***Heteroxenia*** |  |  |  |  |
| *H. elizabethae* | CASIZ 184573 | KJ511378 | KJ511338 | KJ511299 |
| *H. fuscescens* | ZMTAU CO 34118 | GQ342462 | GQ342528 | JX203756 |
| *H. mindorensis* | CASIZ 184566 | KJ511379 | KJ511339 | KJ511300 |
| *Heteroxenia* sp. | CASIZ 184553 | KJ511382 | KJ511342 | KJ511303 |
| ***Ovabunda*** |  |  |  |  |
| *O. ainex* | USNM1201935 | KC864922 | KC864858 | xxxxxxxx |
| *O. andamanensis* sp. n. | PMBC 11860 | xxxxxxxx | xxxxxxxx | xxxxxxxx |
| *O. andamanensis* sp. n. | PMBC 11861 | xxxxxxxx | xxxxxxxx | xxxxxxxx |
| *O. andamanensis* sp. n. | PMBC 11862 | xxxxxxxx | xxxxxxxx | xxxxxxxx |
| *O. arabica* | USNM1201939 | KC864926 | KC864862 | xxxxxxxx |
| *O. biseriata* | ZMTAU CO 34077 | GU356004 | GU356027 | KJ511307 |
| *O. biseriata* | USNM1201938 | KC864925 | KC864861 | xxxxxxxx |
| *O. biseriata* | USNM1201943 | KC864930 | KC864866 | xxxxxxxx |
| *O. biseriata* | USNM1201944 | KC864931 | KC864867 | xxxxxxxx |
| *O. biseriata* | USNM1201958 | KC864941 | KC864876 | xxxxxxxx |
| *O. gohari* | USNM1201936 | KC864923 | KC864859 | xxxxxxxx |
| *O. gohari* | USNM1202009 | KC864984 | KC864915 | xxxxxxxx |
| *O. macrospiculata* | USNM1201941 | KC864928 | KC864864 | xxxxxxxx |
| *O. macrospiculata* | USNM1201979 | KC864959 | KC864892 | xxxxxxxx |
| *O. verseveldti* | USNM1202008 | KC864983 | KC864914 | xxxxxxxx |
| ***Sansibia*** |  |  |  |  |
| *Sansibia* sp. | CASIZ 184572 | KJ511384 | NA | NA |
| *Sansibia* sp. | NTM C012955 | NA | DQ302840 | NA |
| ***Sarcothelia*** |  |  |  |  |
| *Sarcothelia sp.* | CSM-CORY1 | xxxxxxxx | xxxxxxxx | xxxxxxxx |
| *Sarcothelia edmondsoni* | CSM-SKB | xxxxxxxx | xxxxxxxx | xxxxxxxx |
| ***Sympodium*** |  |  |  |  |
| *S. caeruleum* | ZMTAU CO 34185 | GU356009 | JX203815 | JX203758 |
| *S. caeruleum* | RMNH Coel. 41526 | KF955271 | NA | KF915548 |
| *S. caeruleum* | USNM1201969 | KC864949 | NA | xxxxxxxx |
| ***Xenia*** |  |  |  |  |
| *X. actuosa* | USNM1201945 | KC864932 | KC864868 | xxxxxxxx |
| *X. fisheri* | CASIZ 184540 | KJ511389 | KJ511349 | KJ511311 |
| *X. hicksoni* | ZMTAU CO 34072 | GQ342463 | GQ342529 | JX203759 |
| *X. kusimotoensis* | CASIZ 184554 | KJ511392 | KJ511352 | KJ511314 |
| *X. lepida* | CASIZ 184562 | KJ511395 | KJ511355 | KJ511317 |
| *X. lillieae* | CASIZ 184533 | KJ511396 | KJ511356 | KJ511318 |
| *X. membranacea* | CASIZ 184536 | KJ511385 | KJ511345 | KJ511308 |
| *X. membranacea* | CASIZ 184546 | KJ511386 | KJ511346 | KJ511309 |
| *X. puerto-galerae* | CASIZ 184532 | KJ511402 | KJ511362 | KJ511324 |
| *X. ternatana* | CASIZ 184560 | KJ511405 | KJ511365 | KJ511327 |
| *X. umbellata* | USNM1202005 | KC864981 | KC864912 | xxxxxxxx |
| *X. viridis* | CASIZ 184542 | KJ511409 | KJ511369 | KJ511331 |
| *Xenia* sp. 1 | CASIZ 184578 | KJ511400 | KJ511360 | KJ511322 |
| *Xenia* sp. 3 | CASIZ 184579 | KJ511401 | KJ511361 | KJ511323 |
| *Xenia* sp. 4 | CASIZ 184561 | KJ511393 | KJ511353 | KJ511315 |
| *Xenia* sp. 5 | CASIZ 184564 | KJ511388 | KJ511348 | KJ511310 |
| *Xenia* sp. 6 | CASIZ 184555 | KJ511406 | KJ511366 | KJ511328 |
| *Xenia* sp. 7 | CASIZ 184557 | KJ511408 | KJ511368 | KJ511330 |
| **Outgroup taxa:** |  |  |  |  |
| ***Coelogorgia palmosa*** | NTM C014914 | GQ342413 | DQ302805 | JX203698 |
| ***Paralemnalia***  ***thyrsoides*** | ZMTAU CO 34087 | GQ342436 | GQ342509 | JX203727 |
| ***Rhytisma fulvum*** | ZMTAU CO 34124 | GQ342396 | GQ342478 | JX203728 |
